# Genetic structure and diversity of indigenous rice (*Oryza sativa*) varieties in the Eastern Himalayan region of Northeast India

**DOI:** 10.1186/2193-1801-2-228

**Published:** 2013-05-19

**Authors:** Baharul Choudhury, Mohamed Latif Khan, Selvadurai Dayanandan

**Affiliations:** Forest and Evolutionary Genomics Laboratory, and Centre for Structural and Functional Genomics, Biology Department, Concordia University, 7141 Sherbrooke St. West, Montreal, Quebec H4B 1R6 Canada; Québec Centre for Biodiversity Sciences, Montréal, QC Canada; Department of Forestry, North Eastern Regional Institute of Science & Technology, NIRJULI, Itanagar, Arunachal Pradesh 791109 India

**Keywords:** Conservation, Eastern Himalaya, Genetic diversity, Genetic structure, Indigenous rice varieties, NE India

## Abstract

The Eastern Himalayan region of Northeast (NE) India is home to a large number of indigenous rice varieties, which may serve as a valuable genetic resource for future crop improvement to meet the ever-increasing demand for food production. However, these varieties are rapidly being lost due to changes in land-use and agricultural practices, which favor agronomically improved varieties. A detailed understanding of the genetic structure and diversity of indigenous rice varieties is crucial for efficient utilization of rice genetic resources and for developing suitable conservation strategies. To explore the genetic structure and diversity of rice varieties in NE India, we genotyped 300 individuals of 24 indigenous rice varieties representing *sali*, *boro*, *jum* and glutinous types, 5 agronomically improved varieties, and one wild rice species (*O. rufipogon*) using seven SSR markers. A total of 85 alleles and a very high level of gene diversity (0.776) were detected among the indigenous rice varieties of the region. Considerable level of genetic variation was found within indigenous varieties whereas improved varieties were monoporphic across all loci. The comparison of genetic diversity among different types of rice revealed that *sali* type possessed the highest gene diversity (0.747) followed by *jum* (0.627), glutinous (0.602) and *boro* (0.596) types of indigenous rice varieties, while the lowest diversity was detected in agronomically improved varieties (0.459). The AMOVA results showed that 66% of the variation was distributed among varieties indicating a very high level of genetic differentiation in rice varieties in the region. Two major genetically defined clusters corresponding to *indica* and *japonica* groups were detected in rice varieties of the region. Overall, traditionally cultivated indigenous rice varieties in NE India showed high levels of genetic diversity comparable to levels of genetic diversity reported from wild rice populations in various parts of the world. The efforts for conservation of rice germplasm in NE India should consider saving rice varieties representing different types with specific emphasis given to *sali* and *jum* types. The protection against the loss of vast genetic diversity found in indigenous rice varieties in NE India is crucial for maintaining future food security in the changing world.

**Electronic supplementary material:**

The online version of this article (doi:10.1186/2193-1801-2-228) contains supplementary material, which is available to authorized users.

## Introduction

The Asian cultivated rice (*Oryza sativa* L.) is one of the most important crops and a major food source for more than half of the global human population. Phylogeographical and archeological evidence suggest that rice was domesticated over 10000 years ago from its wild ancestor *O. rufipogon* in the region south of the Himalayan mountain range, likely in the present day Eastern and NE India, extending Eastward to Nepal, Myanmar and Thailand to Southern China (Chang 1976; Khush 1997; Londo et al. 2006). A recent study suggests that one of the two sub-species of Asian rice, *O. sativa* ssp *indica* was domesticated in Southeast and South Asia while the other sub-species, *O. sativa* ssp *japonica* was domesticated in Southern China (Huang et al. 2012). During the domestication process, individuals with desirable traits have been selected leaving most of the genetic diversity behind in the progenitors (Doebley et al. 2006). Zhu et al. (2007) estimated that the cultivated rice contains only about 25% of the genetic diversity found in its wild progenitors depicting severe genetic erosion during domestication. Furthermore, a considerable level of genetic diversity was lost during the agronomic improvement of commonly cultivated rice.

**Figure 1:**
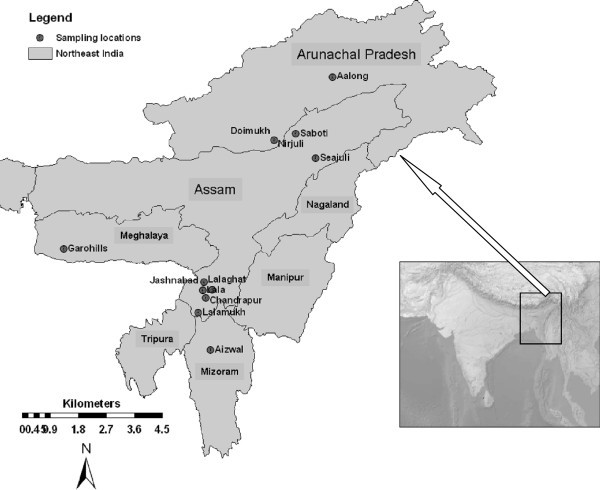
**Map of Northeast India showing sampling sites of traditionally cultivated indigenous rice varieties.**

Studies have shown that indigenous crop varieties traditionally cultivated and maintained by farmers contain high level of genetic diversity and can serve as potential genetic resources for improving yield, resistance to pests and pathogens, and agronomic performance (Brush 1995; Hoisington et al. 1999; Mandel et al. 2011). The Eastern Himalayan region of NE India, a geographical area of over 255,000 km^2^ consisting of Arunachal Pradesh, Assam, Manipur, Meghalaya, Mizoram, Nagaland and Tripura states (Figure 1), is home to a large number of indigenous rice varieties. These varieties are cultivated in diverse topographic and agroclimatic conditions, and normally classified into different types based on the season of cultivation, habitat conditions and the grain quality.

The *sali* type, which comprises majority of rice varieties of the region is cultivated in low-lying flood plains of NE India, mainly in the Brahmaputra and Barak Valley regions. The *boro* type is traditionally cultivated during the winter months (November through May) in low-lying areas where sufficient water is available during the cold and dry months of the year. Thus, *boro* type rice varieties may contain genotypes suitable for cold adaptation. The dryland cultivated rice varieties, normally grown in slash and burn agriculture system, and locally known as *jum* type, show adaptations to a wide range of ecological conditions including low levels of soil moisture in areas at high altitudes reaching over 3000 m above sea level. The glutinous grain type rice is commonly cultivated throughout the region as a source of grain for breakfast and dessert for many ethnic communities in the region. In addition to cultivated indigenous rice varieties, natural populations of many wild rice species including *O. rufipogon*, *O. granulata*, *O. officinalis*, *O. nivara*, *O. meyeriana*, *Hygrorhiza aristata*, *Leersia hexandra* and *Zizenia latifolia* are also found in the Northeastern region of India (Hore 2005).

The indigenous rice varieties cultivated by traditional farmers may contain a considerable genetic diversity that can serve as a source of germplasm for genetic improvements of cultivated varieties of rice. In general, diverse landraces traditionally cultivated by farmers around the centers of diversity and domestication of crops are considered as key natural resources (Pusadee et al. 2009) important for maintaining the future food security in light of the changing climate. Although a few studies have examined the population genetic structure of *O. sativa* germplasm at a global scale (Glaszmann 1987; Garris et al. 2005), region specific studies are limited. Earlier studies based on morphology and agronomic traits (Vairavan et al. 1973; Borkakati et al. 2000; Sarma and Pattanayak 2009) as well as molecular markers (isozyme, RAPD, ISSR) demonstrated a high level of genetic diversity among indigenous rice varieties in NE India (Glaszmann et al. 1989; Sarma and Bahar 2005; Bhuyan et al. 2007). However, these studies were limited either to a particular group of varieties (*e.g.* glutinous rice and lowland varieties) or to a narrow geographic region. In particular, no extensive studies have focused on the genetic structure of some of the widely cultivated indigenous types such as *boro* (cultivated in low-lying perennial water bodies during winter season), *jum* (cultivated in upland areas in hill-slopes and low soil moisture condition), *sali* (most widely cultivated rice during monsoon season) and glutinous (sticky rice with cultural importance) covering the wider geographic area.

The ongoing rapid changes in agricultural practices that favor agronomically improved varieties has become a serious threat for the persistence of indigenous rice varieties in NE India. Thus, conservation and management strategies are urgently needed to prevent further loss of genetic diversity inherent to indigenous rice varieties in the region. A detailed understanding of the genetic structure and diversity is needed for the planning and implementation of effective conservation, management and utilization of rice germplasm in the whole region.

The objectives of the present study are to (1) assess genetic diversity among indigenous rice varieties in the Eastern Himalayan region of the NE India, (2) compare the genetic diversity in indigenous varieties with agronomically improved varieties (3) assess distribution of genetic diversity among different types and (4) infer the population genetic structure of rice varieties in NE India.

## Materials and methods

### Plant samples

A total of 29 varieties of cultivated rice (*Oryza sativa*) were collected from various regions of NE India (Figure 1). These samples included 24 indigenous varieties representing *sali* (12), *jum* (4), *boro* (3), and glutinous (5) types and 5 agronomically improved varieties. The variety name, type and locality are given in Table 1. Wild rice (*O. rufipogon*) accessions originally collected from Eastern India were obtained from the International Rice Research Institute (IRRI), Philippines. Either grains or fresh leaf samples were collected from the field and morphological characters were noted based on direct observation or interviewing the farmers. The agronomically improved varieties, released by the regional and central rice research institutes and widely cultivated for their higher yield were obtained from farmers of the region. Seeds were germinated in Petri dishes and transferred to small pots and grown in a greenhouse. Leaf samples from seedlings were harvested, air dried, and used for the study. Genomic DNA was extracted following a modified cetyltrimethyl ammonium bromide extraction protocol (Doyle and Doyle 1987; Dayanandan et al. 1997).

**Table 1:** **Cultivation type, location and genetic diversity values of traditionally cultivated indigenous and agronomically improved rice varieties including the wild rice (*O. rufipogon*) in Northeast India (AP, Arunachal Pradesh; AS, Assam, ML, Meghalaya, MZ, Mizoram)**

Variety name	Type	Location	A	Na	Npo	Npe	R_A_	***I***	***H***_e_
Lahi	Sali	Doimukh (AP)	11	1.571	3	42.86	1	0.271	0.187
Local Basmati	Sali	Doimukh (AP)	9	1.286	2	28.57	-	0.148	0.105
Borjahinga	Sali	N. Lakhimpur, (AS)	10	1.429	2	28.57	-	0.187	0.130
Joha	Sali	Doimukh (AP)	8	1.143	1	14.29	-	0.096	0.076
Hati Hali	Sali	N. Lakhimpur, (AS)	13	1.857	5	71.43	1	0.377	0.263
Balam	Sali	Cachar (AS)	12	1.714	3	42.86	-	0.328	0.222
Lallatoi	Sali	Hailakandi (AS)	23	3.286	6	85.71	4	0.854	0.498
Arfa	Sali	Hailakandi (AS)	13	1.857	4	57.14	1	0.438	0.305
Mulahail	Sali	Hailakandi (AS)	20	2.857	5	71.43	1	0.719	0.435
Guaroi	Sali	Hailakandi (AS)	13	1.857	5	71.43	-	0.330	0.219
Harinarayan	Sali	Hailakandi (AS)	11	1.571	3	42.86	-	0.262	0.166
Bherapawa	Sali	Hailakandi (AS)	8	1.143	1	14.29	-	0.072	0.051
Papue	Jum	West Siang (AP)	9	1.286	2	28.57	-	0.143	0.105
Sorpuma	Jum	Doimukh (AP)	10	1.429	3	42.86	-	0.239	0.181
Kawanglawang	Jum	Aizwal, (MZ)	17	2.429	6	85.71	1	0.578	0.365
Mimutim	Jum	Garo Hills (ML)	17	2.429	5	71.43	3	0.595	0.384
Til Bora	Glutinous	N. Lakhimpur, (AS)	12	1.714	5	71.43	-	0.237	0.152
Kakiberoin	Glutinous	Hailakandi (AS)	12	1.714	4	57.14	-	0.306	0.207
Borua Beroin	Glutinous	Cachar (AS)	14	2.000	4	57.14	-	0.357	0.224
Ranga Borah	Glutinous	N. Lakhimpur, (AS)	13	1.857	3	42.86	1	0.239	0.135
Bas Beroin	Glutinous	Cachar (AS)	10	1.429	3	42.86	-	0.288	0.228
Aubalam	Boro	Cachar (AS)	15	2.143	5	71.43	1	0.569	0.394
Bashful	Boro	Cachar (AS)	11	1.571	3	42.86	-	0.315	0.232
Moircha	Boro	Cachar (AS)	11	1.571	3	42.86	-	0.167	0.098
Ranjit	Improved	Hailakandi (AS)	7	1	0	0	-	0	0.000
IR8	Improved	Hailakandi (AS)	7	1	0	0	-	0	0.000
Bahadur	Improved	Hailakandi (AS)	7	1	0	0	-	0	0.000
Pankaj	Improved	Hailakandi (AS)	7	1	0	0	-	0	0.000
Joya	Improved	Hailakandi (AS)	7	1	0	0	-	0	0.000
*O. rufipogon*	Wild	Eastern India	29	4.833	6	85.71	4	1.137	0.556

A = Observed no. of allele; Na = Average no. of alleles per 7 loci; Npo = No. of polymorphic loci; Npe = Percent polymorphic loci; R_A_ = Rare allele; I = Shannon information index; *H*e = Nei gene diversity.

### PCR assay and genotyping

Seven SSR loci (RM302, RM341, RM130, RM307, RM169, RM204, RM264) with relatively high polymorphism and distributed across the rice genome were selected for the genetic diversity analyses (Table 2) (Chen et al. 1997; Temnykh et al. 2000). The forward primers were labeled with IRD700 or IRD800 dye for genotyping in LI-COR 4000 IR2 DNA analyzer (Li-Cor Biosciences, Lincoln, NE). The PCR amplifications were performed in 25 μL reaction mixture consisting of 0.2 mM dNTP, 2.5 mM MgCl_2_, 2.5 μL of 10× buffer, 2.5 pmol of each primer and 0.2 U *Taq* polymerase. The thermocycling profile used was initial denaturation at 94° (3 min) followed by 35 cycles of 94° (2 min), 50° (1 min), 72° (2 min) and a final extension of 72° for 5 min. The amplified products were diluted (1:50) with loading dye (Formamide and Bromophenol blue), denatured at 94°C for 5 min and cooled on ice before loading to 6.0% denaturing polyacrylamide gels on a Li-COR automated DNA sequencer with a size standard (50–350 bp, IRDye700 or IRD-800) (Li-Cor Biosciences).

**Table 2:** **Details of SSR loci used in the present study and their genetic diversity parameters**

Primer name	Chr	SSR motif	Forward 5-3	Reverse 5-3	***N***a	***H***_e_
RM302	1	(GT)30(AT)8	TCATGTCATCTACCATCACAC	ATGGAGAAGATGGAATACTTGC	10	0.805
RM341	2	(CTT)20	CAAGAAACCTCAATCCGAGC	CTCCTCCCGATCCCAATC	19	0.861
RM130	3	(GA)10	TGTTGCTTGCCCTCACGCGAAG	GGTCGCGTGCTTGGTTTGGTTC	4	0.419
RM307	4	(AT)14(GT)21	GTACTACCGACCTACCGTTCAC	CTGCTATGCATGAACTGCTC	9	0.749
RM169	5	(GA)12	TGGCTGGCTCCGTGGGTAGCTG	TCCCGTTGCCGTTCATCCCTCC	14	0.798
RM204	6	CT)44	GTGACTGACTTGGTCATAGGG	GCTAGCCATGCTCTCGTACC	18	0.866
RM264	8	(GA)27	GTTGCGTCCTACTGCTACTTC	GATCCGTGTCGATGATTAGC	21	0.884

Chr, Chromosome location; Na, Observed number of alleles; He, Nei (1973) genetic diversity.

The size of each amplified fragment was determined by comparison with the size standard and scored to prepare the genotype matrix. To determine the optimum number of individuals per variety to be genotyped to capture the total diversity, the number of individuals analyzed were increased one by one until the number of alleles reached to a maximum with no further increase for a given locus. Accordingly, we determined that 10 individuals per variety was sufficient to capture the total genetic variation in a given variety. Therefore, we genotyped 300 individuals (10 individuals per variety for 30 varieties) at seven SSR loci for the present study.

### Data analysis

The SSR genotype data matrix was used for assessing genetic diversity and structure in a hierarchical manner from overall (all indigenous varieties), through different types and each variety. The among type genetic diversity was calculated by considering all genotyped individuals of a given type as one population while genetic parameters for among variety was calculated based on 10 genotyped individuals per variety. The observed average number of alleles per locus (*N*a), average allelic richness (*R*_S_), population differentiation (*F*_ST_) and Nei gene diversity (*H*e) (Nei 1973) were calculated using FSTAT 2.9.2.3 (Goudet 2001). Allelic richness is the number of alleles for each population averaged over loci and standardized for the smallest population size. Average effective number of alleles (*N*e) and Shannon information index (*I*) were calculated using PopGene version 1.31 (Yeh et al. 1999). Average pairwise genetic differences between varieties was calculated using Arlequin 3.5 (Excoffier and Lischer 2010). Analysis of Molecular Variance (AMOVA) (Excoffier et al. 1992) within variety, among variety and among types was performed in Arlequin 3.5 (Excoffier and Lischer 2010) to determine the distribution of variation at different hierarchical levels. The statistical significance of the variance components was tested with 1000 permutations.

Genetic distance among varieties were estimated using chord genetic distance method (Cavalli-Sforza and Edwards 1967). The genetic distance based clustering was performed with the unweighted pairgroup method with arithmetic mean (UPGMA) using PowerMarker v3.25 (Liu and Muse 2005), and the dendrogram was constructed using MEGA software (Kumar et al. 2001). Principal component analysis (PCA) of pairwise genetic distance between individuals was performed using GenALEx v. 6.4 (Peakall and Smouse 2006). The Bayesian model-based clustering analysis was used for determining the optimal number of genetic clusters found among rice varieties using the software STRUCTURE 2.3.3 (Pritchard et al. 2000), which partitions individuals into number of clusters (*K*) based on the multilocus genotypic data. The admixture model and correlated allele frequencies were applied for each run with 10,000 burn-in period (iteration) and 100,000 Markov Chain Monte Carlo (MCMC) replication. The optimum *K* value, which indicates the number of genetically distinct clusters in the data, was determined from 10 replicate runs for each value of *K* (Evanno et al. 2005). The Δ*K* was based on the change in the log probability of the data between successive *K* values. Software program Structure Harvester v6.0 (Earl and von Holdt, 2011) was used for calculating parameters of Evanno et al. (2005). The results of five independent runs were consistently converged to the same values.

## Results

### Overall microsatellite diversity

The seven selected SSR loci amplified DNA fragments from 29 *O. sativa* varieties and *O. rufipogon* with consistent reproducibility. A total of 96 alleles with an average of 13.57 alleles per locus were detected among all studied samples. The highest number of alleles (21) was detected in the locus RM264 and the lowest (4) was in the locus RM130. The indigenous rice varieties were genetically variable, while agronomically improved varieties were monomorphic within varieties at all loci. The highest gene diversity value of 0.884 was detected at RM264 and the lowest value of 0.419 detected in RM130 (Table 2).

Indigenous rice varieties in NE India showed high level of genetic diversity with an overall allelic richness of 10.205 per locus and a gene diversity value of 0.776, while the agronomically improved varieties had significantly lower average allelic richness of 2.857 per locus and gene diversity was 0.459. A very high level of differentiation (*F*_ST_ = 0.754) was also detected among the rice varieties.

### Within variety genetic diversity

The average observed number of alleles among indigenous rice varieties ranged from 1.14 (*Joha* and *Bherapawa*) to 3.29 (*Lallatoi*) while the corresponding value was only 1.00 for the agronomically improved varieties. Some of the elite traditional rice varieties (including *Lallatoi*, *Mulahail*, *Aubalam*, *Mimutim*) showed very high levels of genetic diversity as measured in average numbers of alleles, rare alleles and Nei gene diversity. Two of those varieties exhibited relatively high numbers of rare alleles (*Lallatoi* = 4; *Mimutim* = 3). Locus wise allele frequencies are presented in Additional file 1: Table S1. Nei’s gene diversity values ranged from 0.051 (*Bherapawa*) to 0.498 (*Lallatoi*) with an average of 0.223 across all indigenous varieties. Shannon information content varied widely across varieties from 0.072 (*Bherapawa*) to 0.854 (*Lallatoi*) and the average was 0.338 across varieties. The diversity parameters across varieties are presented in Table 1. The pairwise genetic differentiation among varieties (*F*_ST_) ranged from 0.375 to 1.000 and highly significant (*p* < 0.001). The pairwise *F*_ST_ values are given in Additional file 2: Table S2.

### Genetic diversity among types

Different levels of genetic variation were observed in different types of indigenous rice from NE India. The highest diversity was detected among the *sali* type with an average allelic richness and gene diversity of 7.585 (±3.604) and 0.747 (±0.127) respectively. The next level of genetic diversity was detected among the *jum* type followed by the glutinous and *boro* types (Table 3). On the other hand, agronomically improved types showed the lowest levels of diversity (average allelic richness 2.798 ± 1.438; average gene diversity 0.459 ± 0.251). All types showed very high inbreeding coefficient ranging from 0.936 to 1.000, which could be attributable to the selfing mating system of the cultivated rice. Among indigenous rice varieties, the highest average gene diversity within type (*H*_S(W)_) was observed in *jum* (0.259) and the lowest was in glutinous type (0.189). Population differentiation study within different types showed very low *F*_ST_ values ranging from 0.023 in *sali* type to 0.036 in *boro* type (Table 3). The AMOVA results showed statistically significant differentiation (p < 0.01) with 25% variation among individuals, 66% among varieties and 9% among cultivation types (Table 4).

**Table 3:** **Population structure and F-statistics of different types of indigenous and agronomically improved rice varieties in NE India**

Type	Allelic richness	Gene diversity	Inbreeding coefficient	***H***_S(W)_	***F***_ST(W)_
Sali	7.585 (3.604)	0.747 (0.127)	0.984	0.222	0.023
Jum	5.056 (3.061)	0.627 (0.187)	1.000	0.259	0.032
Glutinous	4.727 (1.901)	0.602 (0.261)	0.936	0.189	0.029
Boro	3.857 (1.864)	0.596 (0.280)	0.980	0.241	0.036
Improved	2.798 (1.438)	0.459 (0.251)	1.000	0	0.029

Allelic richness is based on minimum sample size of 30 diploid individuals. *H*_S(W)_ = average genetic diversity within type; *F*_ST(W)_ = genetic differentiation within type. Values in parenthesis represent standard deviation.

**Table 4:** **Analysis of molecular variance (AMOVA) based on 7 SSR loci of traditional and agronomically improved rice varieties in Northeast India**

Amova analysis	df	SS	MS	% of variation	P-value
Among type	4	294.45	129.78	8	>0.001
Among varieties	24	912.96	76.54	66	>0.001
Within varieties	270	366.05	2.80	26	>0.001

df, degree of freedom; SS, sum of square; MS, Means of square.

### Genetic structure analysis

**Figure 2:**
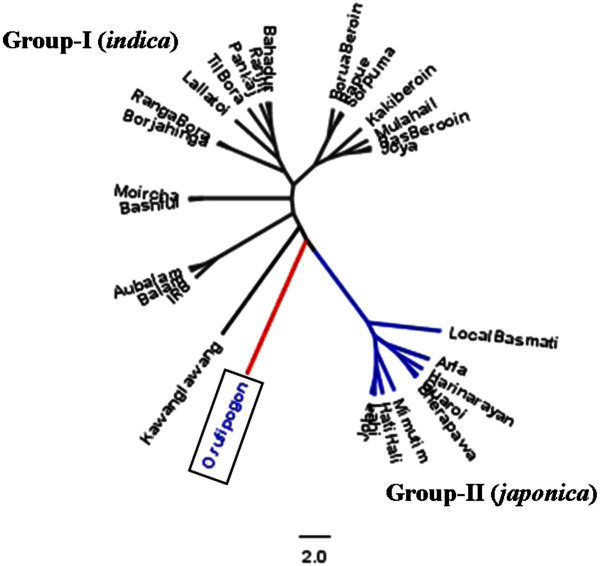
**UPGMA tree based on chord genetic distance (Cavalli-Sforza and Edwards**^1967^**) showing genetic relationships among 29 rice varieties in Northeast India.**

The UPGMA clustering based on chord genetic distance grouped rice varieties into two distinct groups (Figure 2). The Group-I in the UPGMA tree consists of both indigenous and the agronomically improved varieties. All agronomically improved varieties clustered within Group-I, which could be considered as *indica* sub-species. The other group (Group-II) consisted of a few indigenous varieties belonging to *sali* and *jum* types and could be considered as the *japonica* sub-species. *O. rufipogon* accessions appeared intermediate between *indica* and *japonica* groups (Figure 2). This analysis revealed that 62.5% of the traditional rice varieties in Eastern Himalayan region of NE India are of sub-species *indica* while 37.5% are *japonica* sub-species.

The UPGMA tree revealed that rice varieties clustered into smaller sub-groups based on type, grain qualities or geographic origin. For example, *boro*, *jum*, glutinous, and agronomically improved varieties clustered together into smaller sub-groups within Group-I (*indica*) while the Group-II (*japonica*) formed two sub-groups corresponding the geographic locations (Additional file 3: Figure S1). A few sub-groups and varieties (marked with double asterisk), however, did not cluster with respective types or grain quality (Additional file 3: Figure S1).

**Figure 3:**
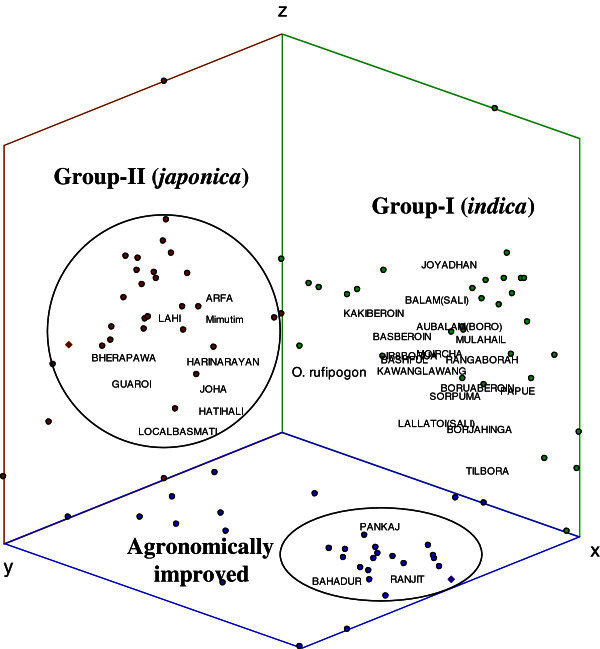
**Principal component analysis of indigenous and agronomically improved rice varieties based on 7 SSR loci.** Different varieties grouped together corresponding to two sub-species (*indica* and *japonica*).

The PCA analysis using pairwise genetic distances revealed that the first three principal components explained 59.91% of the total variation and showed similar clustering of rice varieties into Group-I (*indica*) and Group-II (*japonica*) (Figure 3). Three of the agronomically improved varieties (*Pankaj*, *Bahadur* and *Ranjit*) formed a distinct group but showed closer affinity to the Group-I (*indica*). *O. rufipogon* accessions showed intermediate position between the two groups (Figure 3) similar to clustering in the UPGMA tree.

**Figure 4:**
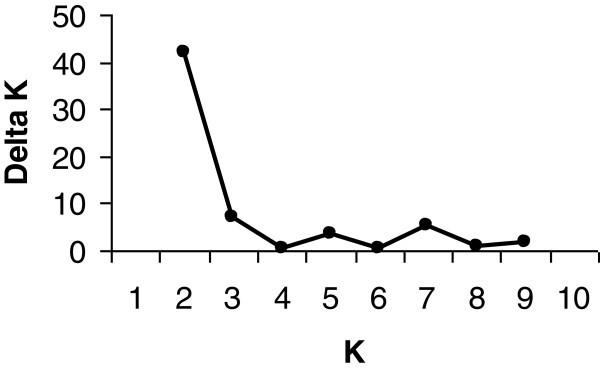
**The relationship between ΔK and K showing the highest peak at K = 2.**

**Figure 5:**
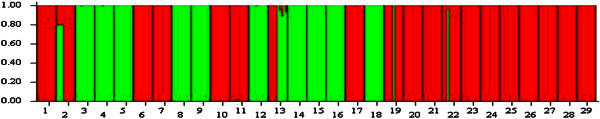
**Population structure of traditionally cultivated indigenous and agronomically improved rice varieties in the Eastern Himalayan region.** The optimal value of K = 2.

The Bayesian based analysis of population structure showed that the highest log likelihood is at *K* = 2 (Figure 4) suggesting two major groups corresponding to two distinct clusters. Individual assignments into two clusters revealed that Group-I (green color, Figure 5) consists of 34% of varieties and include sub-species *japonica* with more than 95% ancestry. The other 52% of varieties including agronomically improved accessions formed Group-II (red color, Figure 5) corresponding to the sub-species *indica* with more than 95% ancestry. However, 14% of the indigenous varieties showed mixed ancestry of both *indica* and *japonica* types. The comparison of STRUCTURE results with UPGMA and PCA results revealed that three varieties (*Kawanglawang*, *Local Basmati* and *Bashful*; varieties 3, 6, and 18 marked with asterisk; Additional file 3: Figure S2a) interchanged between Group-I (*indica*) and Group-II (*japonica*). However, independent STRUCTURE runs without agronomically improved varieties grouped these varieties into the groups concordant with UPGMA and PCA analyses (Additional file 3: Figure S2b). Thus, the results of model based STRUCTURE analysis is in agreement with the UPGMA and PCA based clustering and grouping of rice varieties is consistent with the classification of *indica* and *japonica* types.

## Discussion

### Genetic diversity

The present study revealed exceptionally high genetic variation, with an average allelic richness of 10.205 and an overall Nei’s gene diversity of 0.776 among indigenous rice varieties in NE India as compared to significantly low average allelic richness (2.798) and gene diversity (0.459) in agronomically improved types. The levels of genetic diversity were also variable across different varieties and much higher than the agronomically improved varieties (Table 1). Although the varieties represent only a sub-set of total rice varieties in the region, the gene diversity detected is higher than the overall gene diversity of rice varieties reported from Yunnan province in China (0.706) (Tu et al. 2007) and Indonesia (0.68) (Thomson et al. 2007). The gene diversity detected in our study is comparable to the overall gene diversity of wild rice *O. rufipogon* (0.77) and *O. nivara* (0.64) populations of the Vientiane Plain of Laos (Kuroda et al. 2007) and the gene diversity of *O. rufipogon* in China (0.670) (Gao 2004). A previous study based on allozyme markers revealed a moderate genetic variability (Nei gene diversity = 0.341) among 289 rice varieties from NE India (Glaszmann et al. 1989). The higher gene diversity values detected in the present study could be attributable to high resolving power of microsatellite markers.

The present study revealed several indigenous rice varieties with high genetic diversity, which includes *Lallatoi*, *Mulahail*, *Aubalam* and *Mimutim* (Table 1). Despite the low yield, the traditional farmers in Hailakandi area (Barak Valley region of Assam) have been cultivating *Lallatoi*, *Mulahail* and *Aubalam* for over many generations presumably for its superior nutritional quality and better taste (personal communication). The local tribal group members in the Garo Hills of Meghalaya pointed out the superior agronomical qualities of *Mimutim.* Our study revealed high genetic diversity in *Mimutum*, one of the highly valued rice varieties by native tribal groups. This reflects the importance of traditional knowledge in evaluation and conservation of indigenous crop genetic resources (Brush and Meng 1998).

Most of the indigenous rice varieties are maintained and cultivated by traditional farmers in narrow geographic regions. However, traditional farming practices are in decline due to preference for agronomically improved varieties for higher yield. Therefore, appropriate conservation measures should be taken to promote the cultivation of indigenous varieties with local traditional knowledge.

The genetic diversity maintained in a species is considered as a function of its ecological and evolutionary history (Hamrick and Godt 1996). The high genetic diversity among NE Indian rice varieties have been described in relation to morpho-physiological characters (Vairavan et al. 1973), enzymatic characters (Glaszmann et al. 1989), agro-morphological traits (Borkakati et al. 2000) and molecular markers including RAPD (Sarma and Bahar 2005) and ISSR (Bhuyan et al. 2007). The high genetic diversity among rice varieties in the NE Indian region could be attributable to combined effect of wide eco-geographical conditions, diverse agro-ecosystems associated with various rice farming practices and diverse human cultural preferences. High genetic diversity is also reported for other crop plants such as *Zingiber officinale* (Sajeev et al. 2011), Chilli (Yumnam et al. 2012), *Curcuma* species (Das et al. 2011), *Citrus* species (Hazarika 2012) commonly cultivated in NE India, highlighting the importance of the region for germplasm conservation of many crop plants.

We compared the levels of genetic diversity among different types of rice cultivated in NE India, and found that *sali* type possessed the highest gene diversity value of 0.747 and average allelic richness of 7.585. The majority of *sali* varieties are maintained by traditional farmers for specific traits such as aroma, grain size and shape, and tolerance to drought, insects and pests, which may contribute to the maintenance of high genetic variation. *Jum* type also showed high level of heterogeneity with gene diversity of 0.627 and average allelic richness of 5.056. The traditional farming systems and local environment associated with adaptation to diverse conditions including water deficient habitats on the slopes of hilly regions may have contributed to the maintenance of high genetic variability among the *jum* type. Due to their inherent high genetic diversity, *sali* and *jum* types should be prioritized in conservation and management plans and future breeding programs.

The high *F*_IS_ values among rice varieties of the region could be due to predominantly selfing breeding system with a very low outcrossing in *O. sativa* species (Oka 1988). The *F*_ST_ results (Table 3) are also supported by AMOVA (Table 4) which indicated that 66% of the total variation was due to differentiation among varieties. This indicates that rice varieties of the Eastern Himalayan region are highly differentiated.

### Population structure

The UPGMA analyses using genetic distance data clustered rice varieties into two groups, which corresponded to *O. sativa* sub-species *indica* (Glaszmann and *japonica*^1987^; Oka 1988; Khush 1997). These results agree with the previous isozyme data based finding that showed the occurrence of two major groups of rice varieties in NE India (Glaszmann 1987). The PCA analysis and Model-based clustering method implemented in the STRUCTURE software also suggested the existence of two major groups corresponding to *indica* and *japonica* sub-species. The majority of varieties including agronomically improved rice varieties clustered as one group within the sub-species *indica*. Most of the varieties were grouped into *indica* sub-species cluster while few varieties clustered into *japonica* sub-species. Vairavan et al. (1973) also reported similar results on the basis of amylose content, agronomic, and morphological characteristics. Our findings were similar to the study involving Indonesian landraces where 68% of the varieties were assigned as *indica* and 32% as *japonica* (Thomson et al. 2007). However, a study of European rice collection revealed that 89% of the accessions belonged to *japonica* type (Courtois et al. 2012). The *O. rufipogon* showed intermediate position between *indica* and *japonica* types suggesting a possible common ancestry of both *indica* and *japonica* types.

Although there was no clear differentiation among *jum*, *sali*, *boro*, and glutinous varieties in the UPGMA and STRUCTURE analysis, the PCA analysis separated the agronomically improved varieties into a distinct group (Figure 3) closely associated with the *indica* type. This is expected as agronomically improved varieties included in the present study were derived from *indica* type. The STRUCTURE analysis did not show evidence for admixture between the *indica* and *japonica* types in almost all varieties. This could be attributable to predominantly selfing or autogamous nature of the breeding system and associated restricted gene flow among populations. Only a few varieties showed mixed ancestry of *indica* and *japonica* type (Figure 5), which may be either due to partial differentiation or rare introgression between the two types. Similar structuring reported among Asian cultivated rice *Oryza sativa* could be due to partial sharing of their ancestral genetic polymorphism and/or recent gene flow (Gao and Innan 2008).

Glaszmann et al. (1989) identified seven groups using isozyme markers and reported typical *indica* and *japonica* sub-species, suggesting that varieties mostly grown in mountainous areas of Meghalaya and Arunachal Pradesh belong to *japonica*. However, the present study revealed that varieties in the mountainous areas of Meghalaya and Arunachal Pradesh represent both *japonica* and *indica* types. Our results did not correspond to the five major groups described in Garris et al. (2005).

## Conclusions

In summary, high genetic diversity detected among traditional rice varieties in the Eastern Himalayan region of NE India is comparable to genetic diversity detected in wild rice populations in various parts of the world. Several varieties with high genetic diversity and cultural importance were found in Barak Valley region of Assam and Garo Hills of Meghalaya. The *sali* and *jum* type showed significantly higher levels of genetic diversity compared to agronomically improved types. Rice varieties in NE India clustered into two major groups corresponding to two sub-species, namely *indica* and *japonica*. Our findings highlights the importance of conservation of rice varieties in NE India as a means of preserving genetic diversity to maintain food security in the changing world.

## Electronic supplementary material

Additional file 1: Table S1: Locus wise allele frequencies of each variety based on 10 genotyped individuals. (XLS 36 KB)

Additional file 2: Table S2: Pairwise F_ST_ values among different rice varieties of eastern himalayan region in Northeast India and wild rice (O. rufipogon). (XLS 24 KB)

Additional file 3: Figure S1: Sub-groups of rice varieties within group-I (*indica*) and group-II (*japonica*) based on cultivation type, grain characteristics and geographic origin. **Figure S2.** STRUCTURE output (**a**) including agronomically improved varieties and (**b**) without agronomically improved varieties. Note that three varieties (Kawanglawang, Local Basmati and Bashful; 3, 6, and 18 marked with asterisk) interchanged between group-I (*indica*) and group-II (*japonica*) groups in (**a**) while all varieties of group-I (*indica*) and group-II (*japonica*) found in UPGMA and PCA analysis clustered together in (**b**). (DOC 168 KB)

## References

[CR1] Bhuyan N, Borah BK, Sarma RN (2007). Genetic diversity analysis in traditional lowland rice (*Oryza sativa* L.) of Assam using RAPD and ISSR markers. Curr Sci.

[CR2] Borkakati RP, Borah P, Deka PC, Khush GS, Brar DS, Hardy B (2000). Genetic divergence in photoperiod-insensitive autumn rice germplasm of Northeast India. Advances in Rice Genetics.

[CR3] Brush SB (1995). In situ conservation of landraces in centers of crop diversity. Crop Sci.

[CR4] Brush SB, Meng E (1998). Farmers’ valuation and conservation of crop genetic resources. Genet Resour Crop Evol.

[CR5] Cavalli-Sforza LL, Edwards AWF (1967). Phylogenetic analysis: Models and estimation procedures. Evolution.

[CR6] Chang TT (1976). The origin, evolution, cultivation, dissemination, and diversification of Asian and African rice. Euphytica.

[CR7] Chen X, Temnykh S, Xu Y, Cho G, McCouch SR (1997). Development of a micosatellite framework map providing genome wide coverage in rice. Theor Appl Genet.

[CR8] Courtois B, Frouin J, Greco R (2012). Genetic diversity and population structure in a European collection of rice. Crop Sci.

[CR9] Das A, Kesari V, Satyanarayana VM, Parida A, Rangan L (2011). Genetic relationship of curcuma species from northeast India using PCR-based markers. Mol Biotechnology.

[CR10] Dayanandan S, Bawa KS, Kesseli RV (1997). Conservation of microsatellites among tropical trees (Leguminosae). Am J Bot.

[CR11] Doebley JF, Gaut BS, Smith BD (2006). The molecular genetics of crop domestication. Cell.

[CR12] Doyle JJ, Doyle JL (1987). A rapid DNA isolation procedure for small quantities of fresh leaf tissue. Phytochem Bull.

[CR13] Earl DA, von Holdt BM (2011). STRUCTURE HARVESTER: a website and program for visualizing STRUCTURE output and implementing the Evanno method.

[CR14] Evanno G, Regnaut S, Goudet J (2005). Detecting the number of clusters of individuals using the software STRUCTURE: a simulation study. Mol Ecol.

[CR15] Excoffier L, Lischer HEL (2010). Arlequin suite ver 3.5: A new series of programs to perform population genetics analyses under Linux and Windows. Mol Ecol Resour.

[CR16] Excoffier L, Smouse PE, Quattro JM (1992). Analysis of molecular variance inferred from metric distances among DNA haplotypes: application to human mitochondrial DNA restriction data. Genetics.

[CR17] Gao L (2004). Population structure and conservation genetics of wild rice *Oryza rufipogon* (Poaceae): a region-wide perspective from microsatellite variation. Mol Ecol.

[CR18] Gao L, Innan H (2008). Non-independent domestication of the two rice subspecies, *Oryza sativa* subsp. *indica* and subsp. *japonica*, demonstrated by multilocus microsatellites. Genetics.

[CR19] Garris A, Tai T, Coburn J, Kresovich S, McCouch SR (2005). Genetic structure and diversity in *Oryza sativa* L. Genetics.

[CR20] Glaszmann JC (1987). Isozymes and classification of Asian rice varieties. Theor Appl Genet.

[CR21] Glaszmann JC, Benyayer P, Arnaud M (1989). Genetic divergence among rices from Northeast India.

[CR22] Goudet J (2001). FSTAT, a program to estimate and test gene diversities and fixation indices, version 2.9.3.

[CR23] Hamrick JL, Godt MJ, Avise JC, Hamrick JL (1996). Conservation genetics of endangered plant species. Conservation Genetics: Case Histories from Nature.

[CR24] Hazarika TK (2012). Citrus genetic diversity of north-east India, their distribution, ecogeography and ecobiology. Genet Resour Crop Ev.

[CR25] Hoisington D, Khairallah M, Reeves T, Ribaut JM, Skovmand B, Taba S, Warburton M (1999). Plant genetic resources: what can they contribute toward increased crop productivity?. Proc Natl Acad Sci.

[CR26] Hore DK (2005). Rice diversity collection, conservation and management in northeastern India. Genet Resour Crop Ev.

[CR27] Huang X, Kurata N, Wei X (2012). A map of rice genome variation reveals the origin of cultivated rice. Nature.

[CR28] Khush GS (1997). Origin, dispersal, cultivation and variation of rice. Plant Mol Biol.

[CR29] Kumar S, Tamura K, Jakobsen BI, Nei M (2001). MEGA2: Molecular evolutionary genetics analysis software. Bioinformatics.

[CR30] Kuroda Y, Sato YI, Bounphanousay C, Kono Y, Tanaka K (2007). Genetic structure of three *Oryza* AA genome species (*O. rufipogon*, *O. nivara* and *O. sativa*) as assessed by SSR analysis on the Vientiane Plain of Laos. Conserv Genet.

[CR31] Liu K, Muse SV (2005). POWERMARKER: Integrated analysis environment for genetic marker data. Bioinformatics.

[CR32] Londo JP, Chiang YC, Hung KH, Chiang TY, Schaal BA (2006). Phylogeography of Asian wild rice, *Oryza rufipogon*, reveals multiple independent domestications of cultivated rice, *Oryza sativa*. Proc Natl Acad Sci USA.

[CR33] Mandel JR, Dechaine JM, Marek LF, Burke JM (2011). Genetic diversity and population structure in cultivated sunflower and a comparison to its wild progenitor, *Helianthus annuus* L. Theor Appl Genet.

[CR34] Nei M (1973). Analysis of gene diversity in subdivided populations. Proc Natl Acad Sci USA.

[CR35] Oka H (1988). Indica-Japonica differentiation of rice cultivars. Origin of cultivated rice.

[CR36] Peakall R, Smouse PE (2006). Genealex 6: Genetic analysis in excel. Population genetic software for teaching and research. Mol Ecol Notes.

[CR37] Pritchard JK, Stephens M, Donnelly P (2000). Inference of Population Structure Using Multilocus Genotype Data. Genetics.

[CR38] Pusadee T, Jamjod S, Chiang YC, Rerkasem B, Schaal BA (2009). Genetic structure and isolation by distance in a landrace of Thai rice. Proc Natl Acad Sci USA.

[CR39] Sajeev S, Roy AR, Iangrai B, Pattanayak A, Deka BC (2011). Genetic diversity analysis in the traditional and improved ginger (*Zingiber officinale* Rosc.) clones cultivated in North-East India. Sci Hortic.

[CR40] Sarma BK, Pattanayak A (2009). Rice diversity of Northeast India.

[CR41] Sarma RN, Bahar B (2005). Genetic variation of bora rice (glutinous rice) of Assam as revealed by RAPDs. Plant Genetic Resources Newsletter.

[CR42] Temnykh S, Park WD, Ayres N, Cartinhour S, Hauck N, Lipovich L, Cho YG, Ishii T, McCouch SR (2000). Mapping and genome organization of microsatellite sequences in rice (*Oryza sativa* L.). Theor Appl Genet.

[CR43] Thomson MJ, Septiningsih EM, Suwardjo F, Santoso TJ, Silitonga TS, McCouch SR (2007). Genetic diversity analysis of traditional and improved Indonesian rice (*Oryza sativa* L.) germplasm using microsatellite markers. Theor Appl Genet.

[CR44] Tu M, Lu BR, Zhu Y, Wang Y (2007). Abundant within-varietal genetic diversity in rice germplasm from Yunnan province of China revealed by SSR fingerprints. Biochem Genet.

[CR45] Vairavan S, Siddiq EA, Arunachalam V, Swaminathan MS (1973). A study on the nature of genetic divergence in rice from Assam and Northeast Himalayas. Theor Appl Genet.

[CR46] Yeh FC, Yang R, Boyle T (1999). POPGENE. Version 1.31. Microsoft Window-based Freeware for Population Genetic Analysis.

[CR47] Yumnam JS, Tyagi W, Pandey A, Meetei NT, Rai M (2012). Evaluation of genetic diversity of Chilli landraces from North Eastern India based on morphology, SSR markers and the Pun1 locus. Plant Mol Biol Rep.

[CR48] Zhu Q, Zheng X, Luo J, Gaut BS, Ge S (2007). Multilocus analysis of nucleotide variation of *Oryza sativa* and its wild relatives: severe bottleneck during domestication of rice. Mol Biol Evol.

