# Supplementary material for: Genetic structure and diversity of indigenous rice (Oryza sativa) varieties in the Eastern Himalayan region of Northeast India
Source: Springerplus. 2013 May 19;2:228. doi: 10.1186/2193-1801-2-228 (PMC3667383; doi:10.1186/2193-1801-2-228)
Supplement: Supplementary file 3 — Additional file 3: Figure S1: Sub-groups of rice varieties within group-I (indica) and group-II (japonica) based on cultivation type, grain characteristics and geographic origin. Figure S2. STRUCTURE output (a) including agronomically improved varieties and (b) without agronomically improved varieties. Note that three varieties (Kawanglawang, Local Basmati and Bashful; 3, 6, and 18 marked with asterisk) interchanged between group-I (indica) and group-II (japonica) groups in (a) while all varieties of group-I (indica) and group-II (japonica) found in UPGMA and PCA analysis clustered together in (b). (DOC 168 KB) [file 40064_2013_293_MOESM3_ESM.doc]

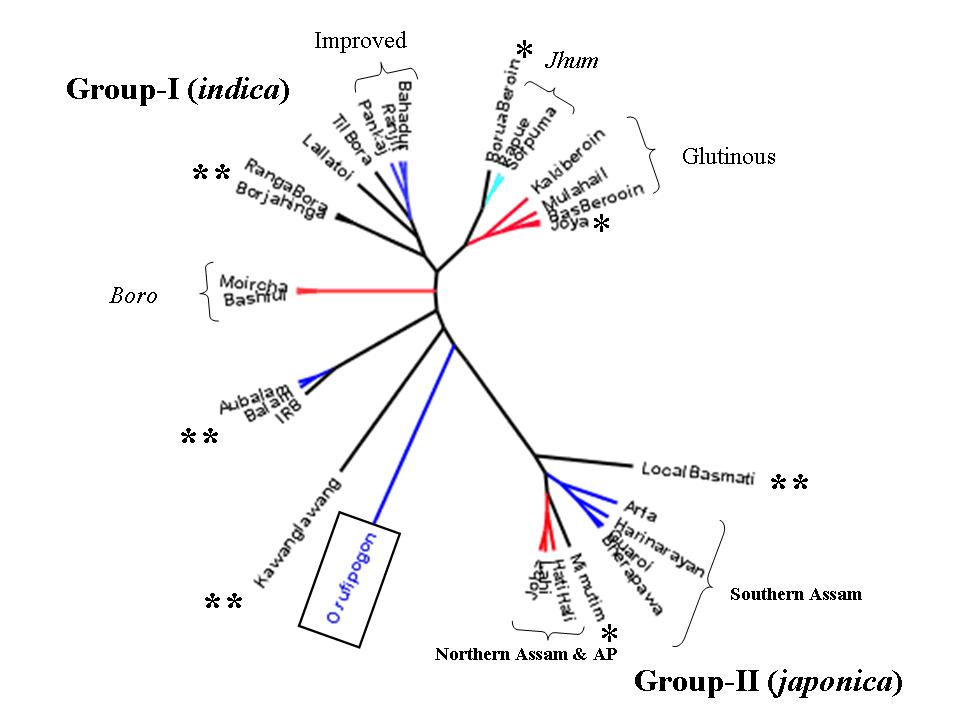


Supplementary Figure S1: Sub-groups of rice varieties within group-I (*indica*) and group-II (*japonica*) based on cultivation type, grain characteristics and geographic origin.


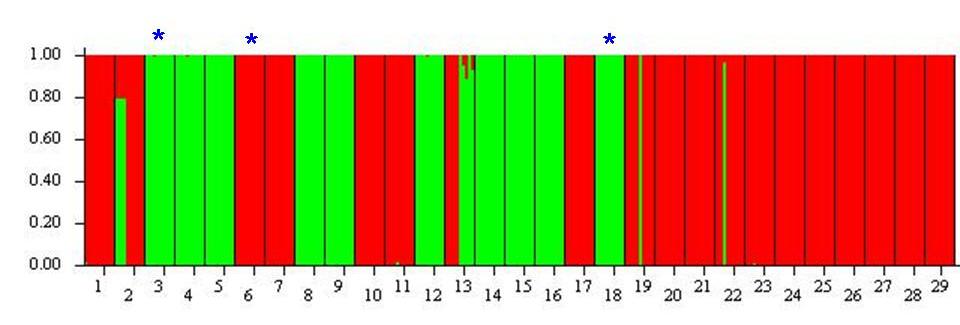
(a)


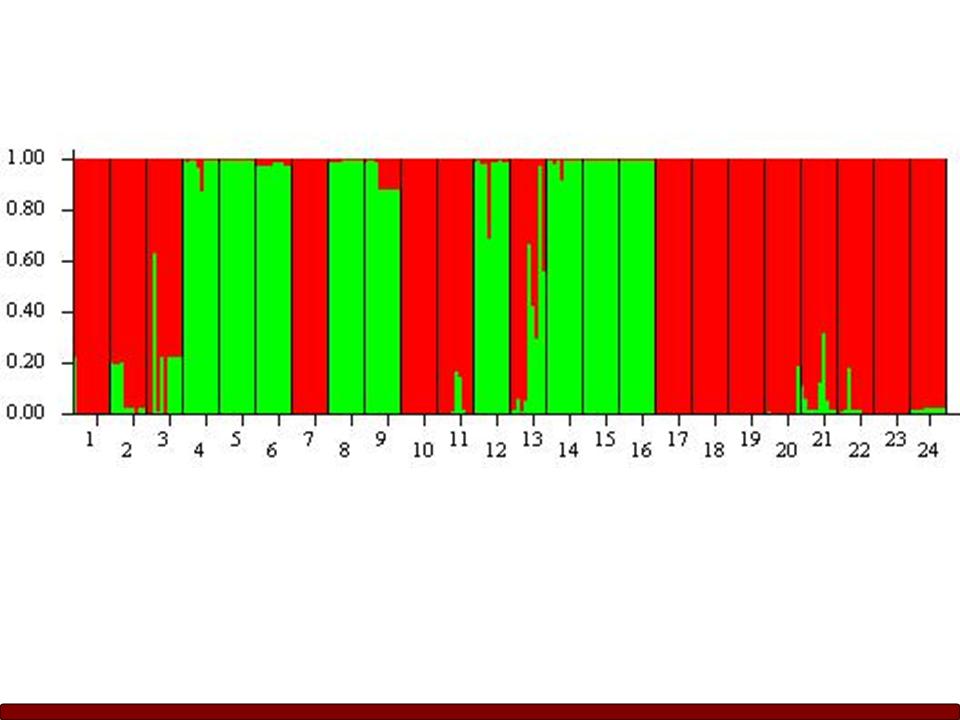


(b)

Supplementary Figure S2: STRUCTURE output (a) including agronomically improved varieties and (b) without agronomically improved varieties. Note that three varieties (Kawanglawang, Local Basmati and Bashful; 3, 6, and 18 marked with asterisk) interchanged between group-I (*indica*) and group-II (*japonica*) groups in (a) while all varieties of group-I (*indica*) and group-II (*japonica*) found in UPGMA and PCA analysis clustered together in (b).
